# The endoplasmic reticulum plays a key role in α-cell intracellular Ca^2+^ dynamics and glucose-regulated glucagon secretion in mouse islets

**DOI:** 10.1016/j.isci.2024.109665

**Published:** 2024-04-05

**Authors:** Samuel Acreman, Jinfang Ma, Geoffrey Denwood, Rui Gao, Andrei Tarasov, Patrik Rorsman, Quan Zhang

**Affiliations:** 1 Oxford Centre for Diabetes, Endocrinology and Metabolism, Radcliffe Department of Medicine, University of Oxford, Oxford OX3 7LE, UK; 2 Institute of Neuroscience and Physiology, Department of Physiology, Metabolic Research Unit, Sahlgrenska Academy, University of Gothenburg, Box 430, S-405 30 Gothenburg, Sweden; 3 Department of Endocrinology and Metabolism, West China Hospital, Sichuan University, Chengdu, China; 4 Biomedical Sciences Research Institute, School of Biomedical Sciences, Ulster University, Coleraine, Northern Ireland, UK; 5 CNC - Center for Neuroscience and Cell Biology, CIBB - Centre for Innovative Biomedicine and Biotechnology, University of Coimbra, Coimbra, Portugal

**Keywords:** Cell biology, Cellular physiology, Physiology

## Abstract

Glucagon is secreted by pancreatic α-cells to counteract hypoglycaemia. How glucose regulates glucagon secretion remains unclear. Here, using mouse islets, we studied the role of transmembrane and endoplasmic reticulum (ER) Ca^2+^ on intrinsic α-cell glucagon secretion. Blocking isradipine-sensitive L-type voltage-gated Ca^2+^ (Ca_v_) channels abolished α-cell electrical activity but had little impact on its cytosolic Ca^2+^ oscillations or low-glucose-stimulated glucagon secretion. In contrast, depleting ER Ca^2+^ with cyclopiazonic acid or blocking ER Ca^2+^-releasing ryanodine receptors abolished α-cell glucose sensitivity and low-glucose-stimulated glucagon secretion. ER Ca^2+^ mobilization in α-cells is regulated by intracellular ATP and likely to be coupled to Ca^2+^ influx through P/Q-type Ca_v_ channels. ω-Agatoxin IVA blocked α-cell ER Ca^2+^ release and cell exocytosis, but had no additive effect on glucagon secretion when combined with ryanodine. We conclude that glucose regulates glucagon secretion through the control of ER Ca^2+^ mobilization, a mechanism that can be independent of α-cell electrical activity.

## Highlights

• Blockade of L-type Ca_v_ channels inhibits pancreatic α-cell electrical activity
• Glucagon secretion can be normal when α-cell electrical activity is abolished
• In α-cells, the ER releases Ca^2+^ in response to glucose metabolism
• Ca^2+^ released from the ER plays a key role in glucagon secretion from the α-cells

## Graphical abstract

**Figure:**
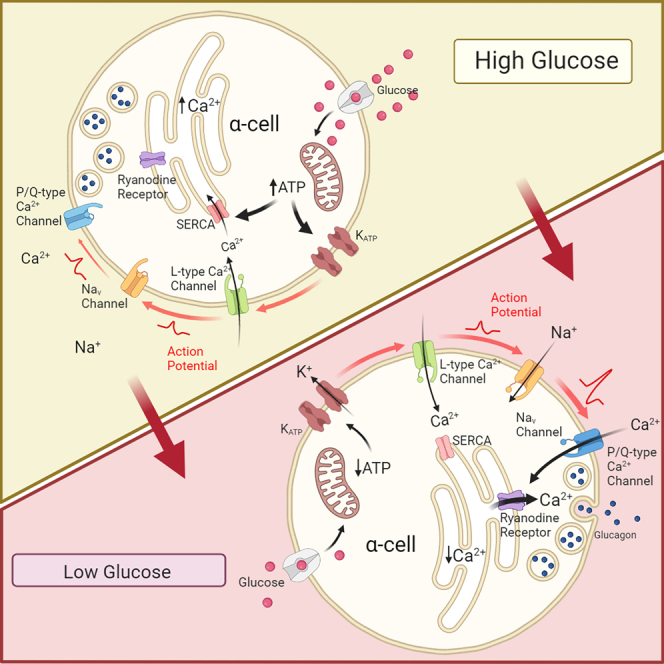

## Introduction

Glucose is the primary cellular fuel and the circulatory concentration is kept within a narrow range. Prolonged high blood glucose (hyperglycaemia) causes severe damage to multiple organs,^1^ while a fall in the glucose level below the normal range (hypoglycaemia) can become a life-threatening condition.^2^ Pancreatic islets play a central role in maintaining glucose homeostasis, secreting the glucose-lowering hormone insulin (from β cells) and glucose-elevating hormone glucagon (from α-cells). The impact of insulin deficiency in diabetes has long been established,^3^ but a causal link between dysregulation of glucagon secretion and the disease has become increasingly recognised.^4^

It is well established that low ambient glucose levels stimulate and eu-, or hyperglycaemic concentrations inhibit glucagon secretion.^5^ However, different from that of β cells, the precise mechanisms by which glucose regulates glucagon secretion remain to be established.^6^^,^^7^ The excitable nature of α-cells^8^ led to the hypothesis that high glucose inhibits glucagon secretion by directly modulating α-cell membrane potential and electrical activity. It has been suggested that glucose controls glucagon secretion via modulation of K_ATP_ channels,^9^^,^^10^ store-operated channels (SOCs),^11^ 2-pore domain K^+^ channels (TWIK-related acid-sensitive K^+^ channel, TASK-1),^12^ or the Na^+^/glucose cotransporter (SGLT2, refs. no.^13^^,^^14^). Some hypotheses suggest opposing effects of glucose on the α-cell membrane potential: either to depolarize via K_ATP_ channel-^10^ or SGLT2-dependent mechanisms^13^^,^^14^; or to repolarize by TASK1-,^12^ SOC-^11^ and fatty acid oxidation-dependent^15^ mechanisms.

These membrane potential-centric mechanisms suggest that glucose modulates α-cell action potential firing frequency and/or amplitude. Changes in electrical activity impact glucagon secretion by altering the activity of voltage-gated Ca^2+^ channels (Ca_v_ channels), which provide Ca^2+^ signals for glucagon granule release through exocytosis. α-cells are equipped with T-, L-, and P/Q-type Ca_v_ channels.^16^ We previously showed that P/Q-type Ca_v_ channel activity governs α-cell exocytosis.^10^ However, in mouse α-cells, P/Q-type Ca_v_ channels only carry a small fraction (∼20%) of Ca^2+^ current, with the majority flowing through L-type Ca_v_ channels^17^ (but this is different in humans^18^). Given that α-cell exocytosis requires an increase in intracellular Ca^2+^,^19^ it is surprising that pharmacological blockade of L-type Ca^2+^ channels (with isradipine or nifedipine) has little effect on either exocytosis or glucagon secretion, in the absence of adrenergic activation.^9^^,^^20^^,^^21^

Changes in intracellular Ca^2+^ can also be modulated by intracellular Ca^2+^ stores. Many organelles can store and release Ca^2+^ in response to cellular signals, such as the mitochondria (see review by Rizzuto et al.*.*^22^), the endo/lysosomal system,^23^ and the endoplasmic reticulum (ER) network. Notably, the ER, as the largest intracellular Ca^2+^ reservoir (100–800 μM luminal free Ca^2+^), can store or release Ca^2+^ according to cytosolic Ca^2+^ [Ca^2+^]_i_ levels and the metabolic state of the cell.^24^ α-cells have an extensive ER network^25^ and Ca^2+^ release from the ER can be triggered without transmembrane Ca^2+^ influx.^11^ Although it was proposed that ER contributes to the regulation of α-cell membrane potential, its relevance to glucose-dependent α-cell exocytosis remains to be established.

In the present study, we dissect the role of L-type Ca_v_ channels and intracellular ER Ca^2+^ stores in the glucose regulation of α-cell cytosolic Ca^2+^ dynamics and glucagon secretion. Our data demonstrate that glucagon secretion can be regulated by an ER-dependent mechanism, independent of α-cell electrical activity. We propose that this novel mechanism may act synergistically with cell electrical activity for precise control of glucagon secretion.

## Results

### L-type Ca_v_ channels are required for α-cell electrical activity in low glucose, but not for glucagon secretion

**Figure 1:**
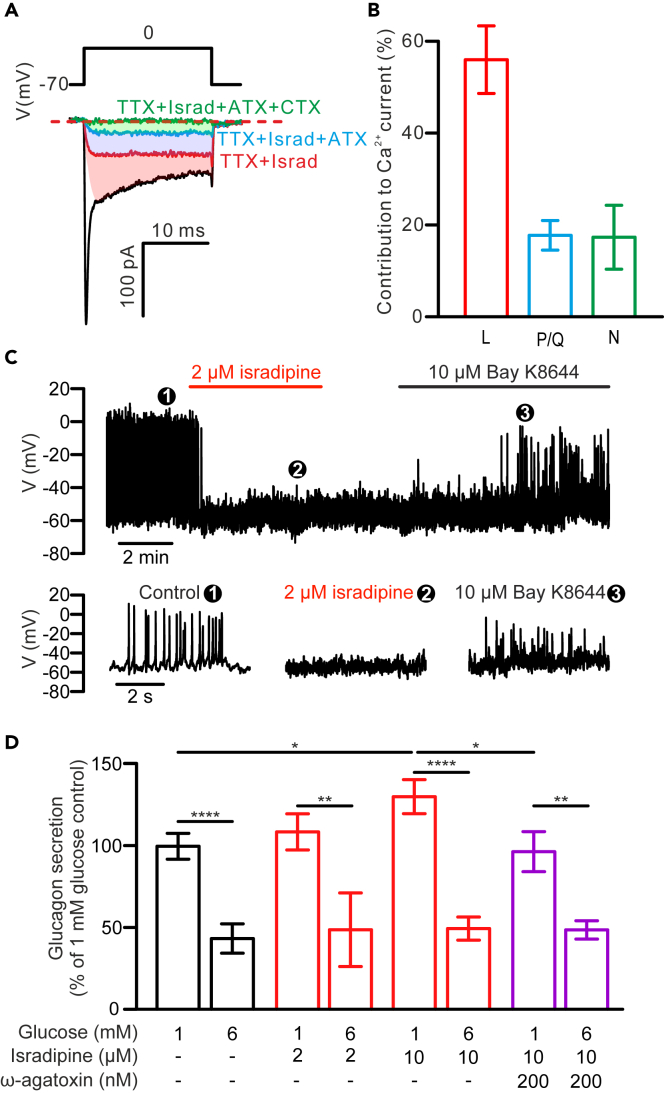
L-type Ca_v_-channels carry the bulk of transmembrane Ca^2+^ and are required for α-cell electrical activity, but not glucagon secretion (A) Depolarisation-triggered Ca^2+^ current recorded in an α-cell, in response to sequential addition of tetrodotoxin (TTX; Na_v_ blocker), isradipine (israd; Red; L-type Ca_v_ channel blocker), ω-agatoxin IVA (ATX; blue; P/Q-type Ca_v_ channel blocker), and ω-conotoxin GIVA (CTX; Green; N-type Ca_v_ channel blocker) as indicated. The shaded areas with respective colors mark the proportion of Ca^2+^ current blocked by the specific blockers. (B) Relative contribution of Ca_v_ channel channels to transmembrane Ca^2+^ current, as measured in (A). L-type Ca_v_ channel, L; P/Q-type Ca_v_ channel, P/Q; N-type Ca_v_ channel, N. (C) Membrane potential recording of an α-cell in the presence of 1 mM glucose. Application of 2 μM isradipine and 10 μM BayK8644 are marked by horizontal bars over the trace. Representative responses under control (❶), 2 μM isardipine (❷) and 10 μM BayK8644 (❸) are displayed below on expanded timescale (bottom). (D) Glucagon secretion from batch-incubated islets in response to 1 and 6 mM glucose in the absence (black bars, *n* = 11) or presence of 2 μM (*n* = 4) or 10 μM (*n* = 11) isradipine (red bars) alone or in combination with 200 nM ω-agatoxin IVA (purple bars; *n* = 7). Data are normalized to secretion at 1 mM glucose in the control and presented as mean values ±SEM. ∗*p* < 0.05, ∗∗*p* < 0.01, and ∗∗∗∗*p* < 0.0001 for indicated comparisons.

Pancreatic α-cells are equipped with several types of Ca_v_ channels that can have distinct roles in cell excitability and exocytosis. First, pharmacological dissection of the α-cell depolarization-triggered Ca^2+^ current was performed using specific Ca_v_ channel blockers, with α-cells identified by their electrophysiological fingerprint.^26^ Under the standard whole-cell voltage-clamp configuration, a 20 ms depolarization from −70 to 0 mV triggered large inward Ca^2+^ currents in α-cells (not blocked by TTX, Figure 1A; −114 ± 15 pA; *n* = 5). Application of the L-type Ca_v_ channel blocker isradipine (2 μM) inhibited 56 ± 7% of the depolarization-triggered Ca^2+^ currents (*n* = 5). In contrast, 17 ± 7% and 18 ± 3% of α-cell Ca^2+^ currents are sensitive to ω-conotoxin GVIA (100 nM), an N-type Ca_v_ channel blocker, and ω-agatoxin IVA (*n* = 5), a P/Q-type Ca_v_ channel blocker (200 nM), respectively (Figure 1B).

Given that the majority of the α-cell Ca^2+^ current is conducted through L-type Ca_v_ channels, we tested whether they contribute to α-cell excitability and glucagon secretion. As shown in Figure 1C, in the presence of low glucose (1 mM), α-cells were electrically active and generated overshooting action potentials. Application of isradipine (2 μM) completely abolished action potential firing at low glucose in all three α-cells tested, an effect that was partially reversed by the L-type Ca_v_ channel activator BayK 8644 (10 μM). However, consistent with previous reports on the effect of dihydropyridines,^9^^,^^21^ 2 μM isradipine did not significantly affect low glucose-stimulated glucagon secretion (*p* > 0.6) or the ability of high glucose to inhibit glucagon secretion (Figure 1D). Interestingly, a higher concentration of isradipine (10 μM, the maximal inhibitory concentration for L-type Ca_v_ channels^27^) exerted a stimulatory effect on glucagon secretion at 1 mM glucose (+20%, *p* < 0.05). Furthermore, the ability of ω-agatoxin IVA to inhibit low-glucose-stimulated glucagon secretion^10^ was largely absent in the presence of 10 μM isradipine.

**Figure 2:**
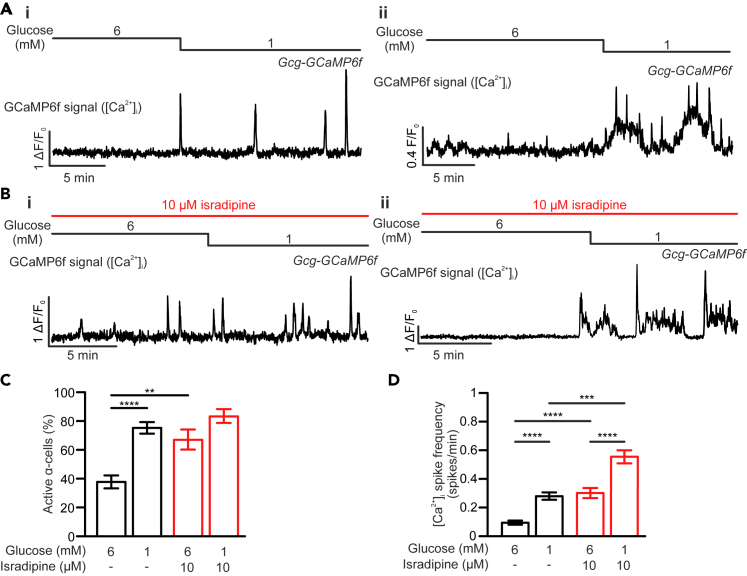
Blockade of L-type Ca_v_-channels does not inhibit α-cell [Ca^2+^]_i_ activity (A) Representative traces demonstrating the α-cell [Ca^2+^]_i_ response, as measured by GCaMP6f from Gcg-GCaMP6f mouse islets, to lowering extracellular glucose from 6 to 1 mM. Two different representative traces included demonstrating fast (i) and slow Ca^2+^ spikes (ii). (B) As in A but shows the responses in the presence of 10 μM isradipine. In A-B, changes in glucose concentration and application of isradipine are indicated by horizontal bars above the traces. (C and D) Bar graphs showing the percentage of active α-cells (C) and [Ca^2+^]_i_ spike frequency (D) in response to the reduction of glucose from 6 to 1 mM in the absence (black bars; *n* = 300 cells from 7 islets) and presence (red bars; *n* = 108 cells from 5 islets) of 10 μM isradipine. Data presented as mean values ±SEM. ∗∗*p* < 0.01, ∗∗∗*p* < 0.001 and ∗∗∗∗*p* < 0.0001 between indicated groups.

The apparent contradiction between cellular excitability and hormone release suggests α-cell intracellular Ca^2+^ dynamics may not be entirely controlled by electrical activity. Using intact Gcg-GCaMP6f mouse islets (Figures S1A and S1B), we next studied glucose-dependent α-cell intracellular Ca^2+^ ([Ca^2+^]_i_) oscillations in the presence and absence of isradipine. Consistent with previous reports,^28^^,^^29^ α-cells can be categorized into two distinct populations based on their [Ca^2+^]_i_ activity, active and inactive, at both low and high glucose concentrations. In the absence of isradipine, when the extracellular glucose concentration was lowered from 6 to 1 mM (Figure 2A), the active α-cell population was doubled (Figure 2C; 76 ± 4% vs. 38 ± 4% of the total α-cells in 6 mM glucose; *p* < 0.001) and the average Ca^2+^ oscillation frequency increased more than 3-fold (Figure 2D; 0.37 ± 0.02 spikes/min vs. 0.10 ± 0.01 spikes/min in 6 mM glucose; *p* < 0.0001). Similar to what was reported recently,^30^ α-cell Ca^2+^ oscillation dynamics were heterogeneous, consisting of fast and/or slow Ca^2+^ transients (Figure 2A). The glucose-dependent response and heterogeneity in α-cell Ca^2+^ activity was largely preserved in the continuous presence of 10 μM isradipine (Figures 2B–2D). Interestingly, when compared with the control, islets treated with isradipine had a larger active population (Figure 2C; *p* < 0.01) and exhibited a 3-fold higher α-cell [Ca^2+^]_i_ activity (Figure 2D; *p* < 0.0001) at 6 mM glucose. Lowering extracellular glucose from 6 to 1 mM remained stimulatory for the α-cell [Ca^2+^]_i_ oscillation frequency (Figure 2D; 0.57 ± 0.04 spikes/min vs. 0.31 ± 0.04 spikes/min in 6 mM glucose; *p* < 0.0001) but only marginally increased the active α-cell population (Figure 2C; from 67 ± 7% to 83 ± 5%; *p* = 0.09). This paradoxical increase in the basal active α-cell Ca^2+^ activity may be attributable to the inhibitory effect of isradipine on intra-islet somatostatin (Figure S2A), a potent paracrine inhibitor of α-cells released by neighboring δ-cells.^31^ Indeed, application of CYN154806 (CYN; 100 nM), a somatostatin receptor inhibitor, stimulated α-cell activity in a manner comparable to that of isradipine (Figures S2B–S2D). While the combination of these two compounds exerted no additive effects on α-cell Ca^2+^ spike frequency (Figures S2E and S2F), isradipine retained the ability to inhibit α-cell electrical activity in the presence of CYN (Figures S3A and S3B). This indicates that the effect of isradipine is independent of α-cell electrical activity and may be attributed to an increase in intracellular cAMP (Figure S3C), similar to that seen with somatostatin receptor blockade, as previously reported.^32^

### Diazoxide and isradipine differentially affect α-cell [Ca^2+^]_i_ activity and intracellular ATP/ADP

**Figure 3:**
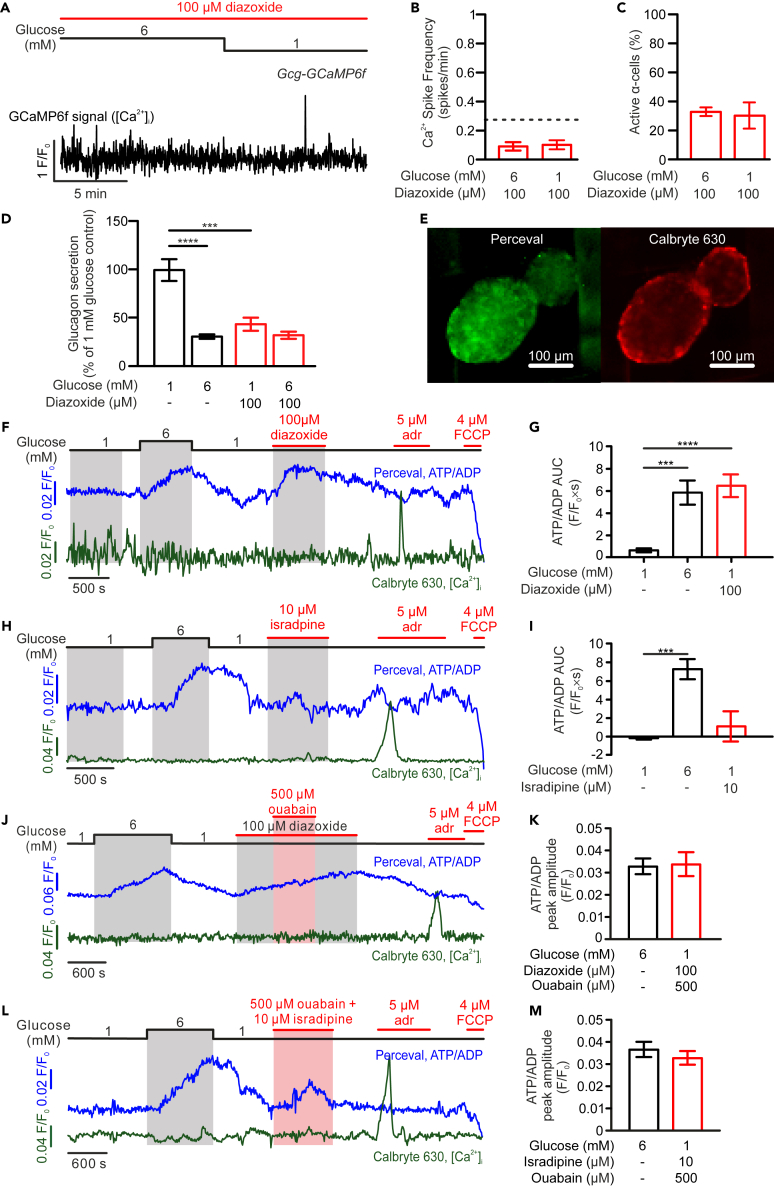
Diazoxide inhibits α-cell Ca^2+^ oscillations and glucagon secretion, but elevates the α-cell intracellular ATP/ADP ratio (A) α-cell [Ca^2+^]_i_ response, as measured by Gcg-GCaMP6f fluorescence, to changes in extracellular glucose from 6 to 1 mM in the continuous presence 100 μM diazoxide. (B) Bar graph summarizing frequency of spontaneous α-cell [Ca^2+^]_i_ spike under the indicated conditions (dashed line represents the frequency value of α-cells at 1 mM glucose alone, as shown in Figure 2C; *n* = 98 cells from 3 islets). (C) As in B but shows the average fraction of active α-cells under the indicated conditions (*n* = 3 islets). (D) Islet glucagon secretion in response to 1 and 6 mM glucose in the absence (black bars; *n* = 5) or presence (red bars; *n* = 5) of 100 μM diazoxide. (E) Image of islets transduced with the ATP/ADP sensor Perceval (left, green) and loaded with Ca^2+^-indicator Calbryte-630 (red, right). (F) Representative traces showing ATP/ADP, as measured by Perceval fluorescence (blue), and Ca^2+^ (green), as measured by Calbryte-630 fluorescence, in an α-cell in response to increasing glucose from 1 to 6 mM and the addition of 100 μM diazoxide. Adrenaline (adr) was used for functional identification of α-cells and FCCP for evaluating the efficiency of Perceval and cell viability. (G) The average AUC of ATP/ADP measured in α-cells under indicated conditions. All the measurements were taken using the same duration, as indicated in the gray shaded areas (F; *n* = 80 cells from 15 islets). (H) As F but shows the response to 10 μM isradipine. (I) As in G but summarizes the α-cell ATP/ADP response to isradipine (*n* = 31 cells from 23 islets). (J) As F but shows the response to the combination of 100 μM diazoxide and 500 μM ouabain. (K) The peak amplitude of ATP/ADP measured in α-cells under indicated conditions (*n* = 15 cells, from 11 islets). (L) As F but shows the response to the combination of 100 μM diazoxide and 500 μM ouabain. (M) The peak amplitude of ATP/ADP measured in α-cells under indicated conditions (*n* = 12 cells, from 10 islets). Data presented as mean ± SEM. ∗∗∗*p* < 0.001 and ∗∗∗∗*p* < 0.001 for indicated comparisons. Horizontal bars in A, F, H, J, and L indicate the duration of the treatments.

The previously data suggest that α-cell [Ca^2+^]_i_ activity and glucagon secretion are not fully dependent on cellular electrical activity. To further test this, we conducted hormone secretion and Ca^2+^ imaging experiments in islets exposed to 100 μM diazoxide, a K_ATP_-channel opener that induces α-cell repolarization.^10^ In contrast with the observations made in the presence of isradipine, 100 μM diazoxide strongly suppressed α-cell [Ca^2+^]_i_ activity and glucagon secretion at low glucose (Figures 3A–3D). Glucose-dependent inhibition of glucagon secretion was shown to involve increases in intracellular ATP.^10^^,^^33^ We next tested whether diazoxide and isradipine, the two antagonists of α-cell electrical activity, exerted differential effects on α-cell intracellular ATP levels. To achieve this, mouse islets were transduced with an adenoviral vector containing Perceval, a fluorescent reporter of the intracellular ATP/ADP ratio.^34^ In addition, a [Ca^2+^]_i_ indicator, Calbryte-630, was also loaded into the same islets, for functional identification of α-cells on the islet periphery—which show a positive [Ca^2+^]_i_ response to adrenaline (Figure 3E). Similar to what was previously reported at high glucose,^35^ application of diazoxide significantly increased α-cell intracellular ATP/ADP ratio at 1 mM glucose, to a level comparable with that at 6 mM glucose, in both intact islets (Figures 3F and 3G) and dispersed single α-cells (Figures S4A and S4B). In contrast to this, 10 μM isradipine did not significantly affect α-cell intracellular ATP/ADP ratio at 1 mM glucose (Figures 3H, 3I, S4C, and S4D). These observations are consistent with a previous report that described an antiparallel relationship between cytosolic Ca^2+^ and ATP oscillations in α-cells.^35^ Furthermore, a large influx of Ca^2+^, triggered by a high concentration of extracellular K^+^, significantly decreased Perceval fluorescence in α-cells, an effect that was dampened by isradipine that reduced high-K^+^-induced Ca^2+^ increase (Figures S4E and S4F).

It is intriguing that the two inhibitors of α-cell electrical activity, which should be equipotent in preventing transmembrane Ca^2+^ influx, exerted disparate effects on α-cell intracellular ATP levels. We reasoned this may be due to their differential effects on α-cell membrane potential. Diazoxide (100 μM) exerts a strong repolarizing effect on α-cells (to ∼ −80 mV),^10^ while isradipine was without significant effect on α-cell membrane potential (∼−50 mV, *cf.* Figure 1C). Cellular membrane potential is maintained by the energy-consuming activity of the Na^+^/K^+^ pump.^36^ Activity of this pump is voltage-dependent and low at hyperpolarized membrane potentials.^37^ Therefore, we tested the effect of Na^+^/K^+^ pump blockade (with ouabain) on cytosolic ATP concentration in islets exposed to isradipine or diazoxide. 500 μM ouabain elevated α-cell intracellular ATP in the presence of isradipine but had no additive effect on islets exposed to diazoxide (Figures 3J–3M; *cf*; Figures 3F–3I). As such, these data suggest that diazoxide increases α-cell ATP via a “sparing effect” caused by slowing down of the Na^+^/K^+^ pump.

### α-cell [Ca^2+^]_i_ oscillations remain sensitive to ATP when the cells are voltage-clamped at resting membrane potential

**Figure 4:**
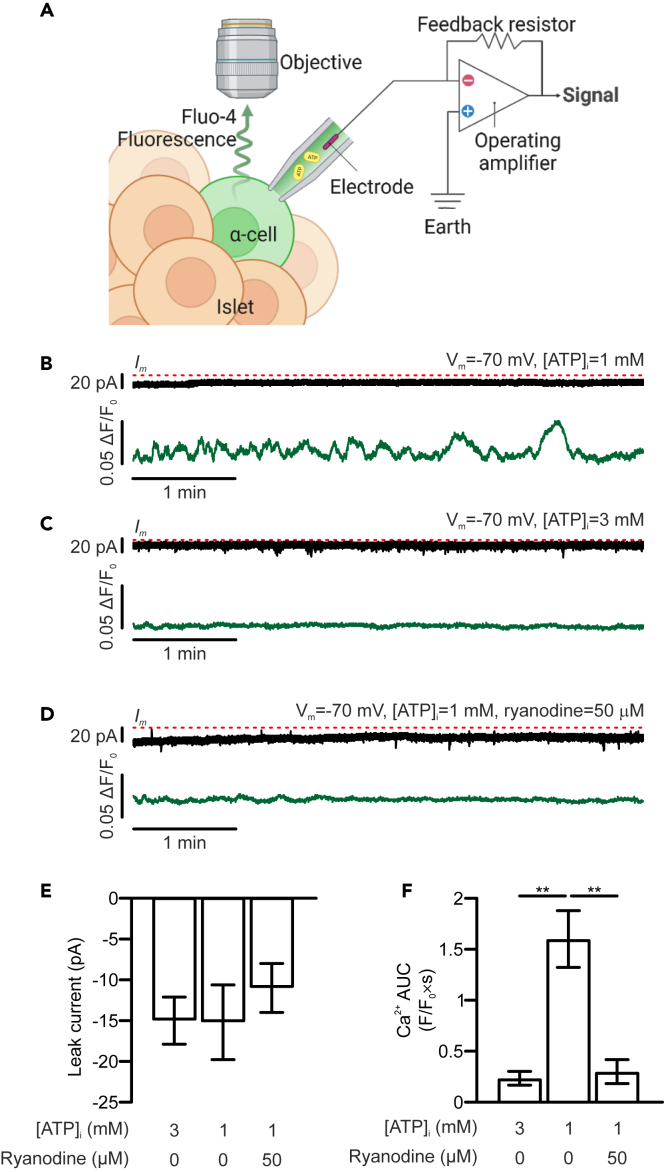
ATP suppresses [Ca^2+^]_i_ oscillations in α-cells voltage-clamped at −70 mV (A) Schematic demonstrating the “patch imaging” experimental setup. A single α-cell within an intact islet is voltage-clamped through a patch-clamping amplifier and a fluorescent Ca^2+^ indicator Fluo-4 (green) is infused into the cell together with ATP (yellow). The α-cell [Ca^2+^]_i_ is reported by changes in the fluorescence of Fluo-4 that is detected through a microscope. (B)–(D) Representative traces of α-cell transmembrane current (top, black) and [Ca^2+^]_i_ oscillations (as measured by Fluo-4 fluorescence, green, bottom) from α-cells voltage-clamped at the resting membrane potential (−70 mV) and infused with 1 mM ATP (B), 3 mM ATP (C) or the combination of 1 mM ATP 50 μM ryanodine (D). Red dashed lines mark the current baselines. (E) Summary of transmembrane current measured in α-cells that were held at −70 mV and infused with ATP and ryanodine with indicated concentrations. (F) Summary of AUC of Ca^2+^ fluorescence measured in α-cells voltage-clamped at −70 mV and intracellularly applied with ATP and ryanodine with indicated concentrations. Data presented as mean ± SEM. *N* = 3 for 3 mM ATP, *n* = 5 for 1 mM ATP and *n* = 3 for 1 mM ATP supplemented with 50 μM ryanodine. ∗∗*p* < 0.01 between indicated groups.

The previous data indicate that intracellular ATP determines α-cell [Ca^2+^]_i_ activity and glucagon secretion, even in the absence of cellular electrical activity. To test this hypothesis, islet α-cell function was interrogated using a combination of electrophysiology and [Ca^2+^]_i_ imaging techniques. As depicted in Figure 4A, α-cells within intact islets were voltage clamped at −70 mV and different concentrations of ATP were applied intracellularly together with a membrane impermeable Ca^2+^ indicator, fluo-4 pentapotassium (Fluo-4). Transmembrane current and [Ca^2+^]_i_ were recorded simultaneously via a patch-clamping amplifier and an sCMOS camera, respectively. Under this experimental condition, no transmembrane Ca^2+^ current was observed in α-cells, irrespective of the concentration of ATP infused (Figures 4B–4E). However, while spontaneous [Ca^2+^]_i_ oscillations were observed in α-cells infused with 1 mM ATP alone, intracellular application of 3 mM ATP or 1 mM ATP supplemented with ryanodine (50 μM) was inhibitory (Figures 4B–4D and 4F).

### The endoplasmic reticulum plays a role in α-cell glucose sensing

**Figure 5:**
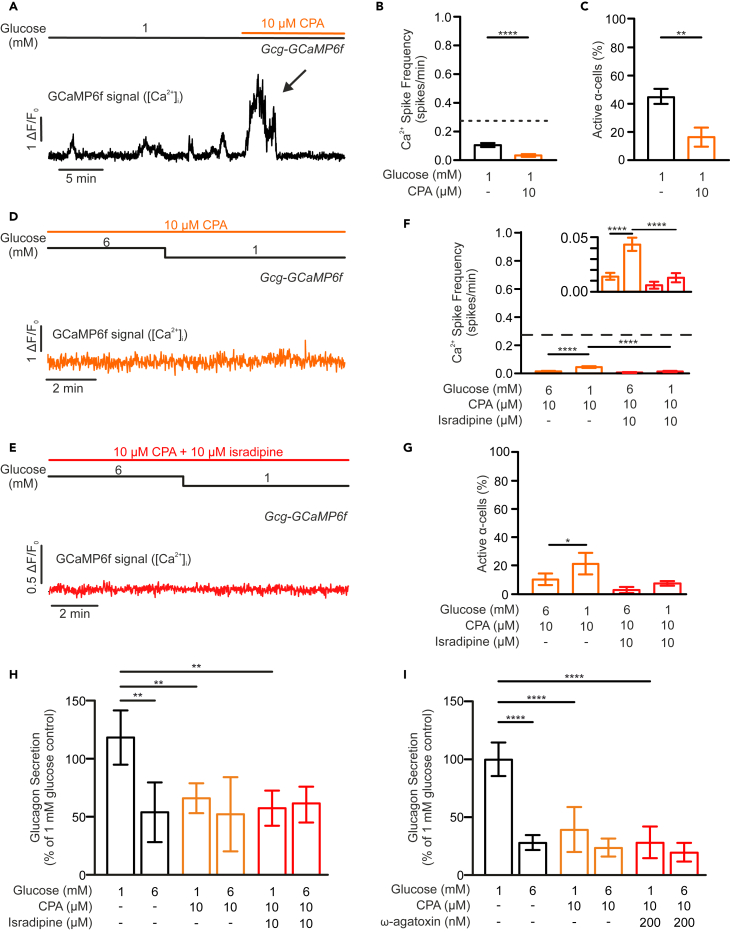
Ca^2+^ release from the ER contributes to α-cell Ca^2+^ activity and is required for low-glucose-stimulated glucagon secretion (A) Gcg-GCaMP6f α-cell [Ca^2+^]_i_ response to 10 μM cyclopiazonic acid (CPA) in the continuous presence 1 mM glucose. The duration of CPA application is marked by the orange horizontal bar. Arrow indicates the initial transient increase in [Ca^2+^]_i_ in response to CPA. (B) Bar graph summarizing frequency of spontaneous α-cell [Ca^2+^]_i_ spike under the indicated conditions (*n* = 109 cells from 3 islets; dashed line represents the frequency value of α-cells at 1 mM glucose alone, as shown in Figure 2C). (C) As in B but shows the average fraction of active α-cells under the indicated condition (*n* = 109 cells from 3 islets). (D and E) Representative traces showing the Gcg-GCaMP6f α-cell [Ca^2+^]_i_ response to a change in the glucose concentration from 6 to 1 mM glucose in the presence of 10 μM CPA (D) and (E) a combination of 10 μM CPA and 10 μM isradipine. (F) Bar graph summarizing frequency of spontaneous α-cell [Ca^2+^]_i_ spike under the indicated conditions (dashed line represents the frequency value of α-cells at 1 mM glucose alone, as shown in Figure 2C). Inset displays the data on an expanded y axis (orange bars mark the effect of CPA alone, *n* = 212 cells from 6 islets; and the red bars show the effect of combining CPA and isradipine, *n* = 138 cells from 4 islets). (G) As in F but shows the average fraction of active α cells under the indicated condition. CPA (orange bars; *n* = 6 islets) or in combination with 10 μM isradipine (red bars; *n* = 4 islets). (H) Islet glucagon secretory response to 1 and 6 mM glucose in the absence (black bars; *n* = 3 for 1 mM glucose, *n* = 4 for 6 mM glucose) or presence (orange bars; *n* = 4 for 1 mM glucose, *n* = 3 for 6 mM glucose) of 10 μM CPA alone or in combination with 10 μM isradipine (red bars; *n* = 3 for 1G, *n* = 4 for 6G). (I) Islet glucagon secretory response to 1 and 6 mM glucose in the absence (black bars; *n* = 4) or presence (orange bars; *n* = 4) of 10 μM CPA alone or in combination with 200 nM ω-agatoxin IVA (red bars; *n* = 4). Data presented as mean ± SEM. ∗*p* < 0.05, ∗∗*p* < 0.01, ∗∗∗*p* < 0.001 and ∗∗∗∗*p* < 0.0001 between indicated groups.

The previous observations suggest a Ca^2+^ source other than transmembrane Ca^2+^ current, through Ca_v_ channels, plays a significant role in glucagon secretion. This Ca^2+^ source is sensitive to cellular metabolic status (as intracellular ATP levels) and contributes toward α-cell metabolic sensing. We hypothesized this is the ER, as it is the cell’s largest intracellular Ca^2+^ store^24^ and its filling is ATP-dependent.^38^ To test this hypothesis, islets were exposed to 10 μM cyclopiazonic acid (CPA), an inhibitor of the sarco/ER Ca^2+^-ATPase (SERCA) pump, which rapidly depletes ER Ca^2+^ (Figures S5A and S5B). At 1 mM glucose, acute application of CPA triggered a transient increase in α-cell [Ca^2+^]_i_ before suppressing [Ca^2+^]_i_ oscillations, reducing both the spike frequency (Figure 5B; *p* < 0.0001) and the number of active cells (Figure 5C; *p* < 0.01). We note that the CPA-induced [Ca^2+^]_i_ transients may reflect Ca^2+^ influx through SOCs, as previously reported,^11^ and was abolished when extracellular Ca^2+^ was replaced with 2 mM Co^2+^ (Figures S5C and S5D).

Interestingly, lowering extracellular glucose from 6 to 1 mM glucose, in the presence of CPA, resulted in an increase in the [Ca^2+^]_i_ oscillation frequency (Figures 5D–5F; see inset for detailed presentation; *p* < 0.0001) and the number of active α-cells (Figure 5G; *p* < 0.05). However, we note that the level of activity remains much suppressed when compared with recordings made in the absence of the SERCA blocker (*cf*. Figure 2C). We hypothesized that the residual [Ca^2+^]_i_ activity may reflect electrical activity-mediated Ca^2+^ influx and would therefore be sensitive to isradipine. Indeed, co-application of CPA and isradipine abolished α-cell [Ca^2+^]_i_ oscillations and low glucose was no longer stimulatory (Figures 5E–5G). Electrical activity-mediated [Ca^2+^]_i_ oscillations alone appeared to be a weak stimulus for the triggering of glucagon release from α-cells, and 10 μM isradipine or 200 nM ω-agatoxin IVA were without additive effect when applied together with CPA. In contrast, CPA alone strongly suppressed low glucose-stimulated glucagon secretion, indicating a significant role played by the ER Ca^2+^ (Figures 5H and 5I).

### The ryanodine receptor is the primary ER Ca^2+^-release channel and opening is triggered by Ca^2+^ influx through the P/Q channel

**Figure 6:**
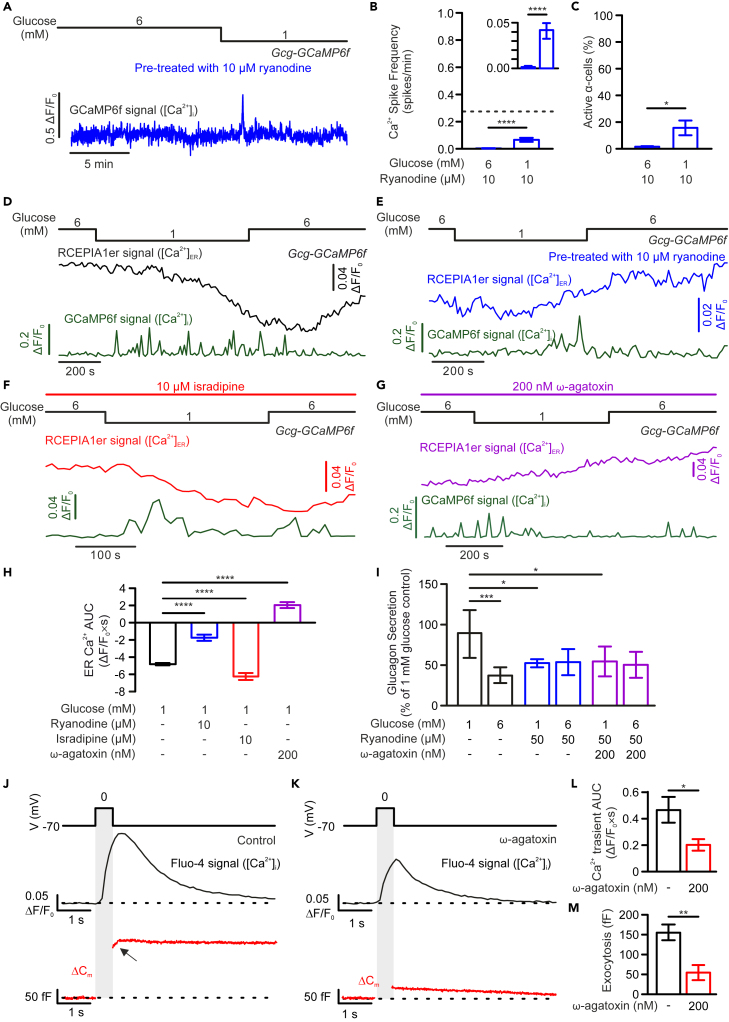
Opening of P/Q Ca_v_ channels triggers ER Ca^2+^ release through ryanodine receptors in α-cells (A) Representative Gcg-GCaMP6f α-cell [Ca^2+^]_i_ response to changes in extracellular glucose from 6 to 1 mM following a 2-h pre-treatment with 10 μM ryanodine. (B) Bar graph summarizing frequency of spontaneous α-cell [Ca^2+^]_i_ spikes under the indicated conditions (dashed line represents the frequency value of α-cells at 1 mM glucose alone, as shown in Figure 2C). Inset displays the data on an expanded y axis (*n* = 183 cells from 5 islets). (C) As in B but shows the average fraction of active α cells following the indicated pretreatment condition (*n* = 5 islets). (D–G) Representative traces showing parallel measurements of α-cell [Ca^2+^]_ER_, measured by RCEPIA1er fluorescence, and [Ca^2+^]_i_, as measured by GCaMP6f fluorescence (green line), to reducing the glucose concentration from 6 to 1 mM in in Gcg-GCaMP6f islets under conditions of (D) control (black, *n* = 87 cells from 15 islets), (E) pre-treatment for 2 h with 10 μM ryanodine (blue, *n* = 46 cells from 11 islets), (F) continuous presence of 10 μM isradipine (red, *n* = 51 cells from 14 islets), and (G) continuous presence of 200 μM ω-agatoxin IVA (purple, *n* = 91 cells from 12 islets). (H) Bar graph summarizing effects of pharmacological treatment of islets on α-cell ER Ca^2+^, as represented in D, E, F, and G, as measured by AUC of RCEPIA1er fluorescence at 1 mM glucose, for the initial 210 s. (I) Glucagon secretion in response to indicated treatment. *N* = 3 for glucose alone (black bars), *n* = 3 for islets pre-treated for 2 h with 10 μM ryanodine (blue bars), *n* = 3 for islets pre-treated for 2 h with 10 μM ryanodine and acutely treated with 200 μM ω-agatoxin IVA (purple bars). (J and K) Examples of α-cell [Ca^2+^]_i_ (ΔF/F_0_; middle; black; measured by Fluo-4 fluorescence) and exocytosis (ΔC_m_; bottom; red; measured by changes in cell capacitance) triggered by depolarizations (from −70 to 0 mV, 500 msl; top), in the absence (Control, J) and presence of 200 nM ω-agatoxin IVA (K). Durations of the depolarization onset are indicated by gray shaded area. Arrow indicates “slow component” of exocytosis. Dashed lines mark the baselines. (L and M) Bar graphs summarizing AUC of α-cell [Ca^2+^]_i_ (L) and exocytosis (M) response to depolarization in the absence (black) and presence of 200 nM ω-agatoxin IVA (red), as in J and K. Data presented as mean ± SEM. ∗*p* < 0.05, ∗∗*p* < 0.01, ∗∗∗*p* < 0.001 ∗∗∗∗*p* < 0.0001 between indicated groups.

As demonstrated previously, Ca^2+^ release from the ER plays a key role in glucagon secretion and α-cell [Ca^2+^]_i_ activity stimulated by hypoglycaemia. We next conducted pharmacological studies to identify the ER-bound Ca^2+^ channels involved in α-cell intracellular Ca^2+^ release. In many cell types, the ER is equipped with two classes of Ca^2+^-releasing channel: ryanodine receptors (RyRs) and inositol triphosphate receptors (IP_3_Rs). In Gcg-GCaMP6f islets pretreated with 10 μM ryanodine, α-cell [Ca^2+^]_i_ activity was reduced to a level similar to that seen with CPA (Figures 6A–6C, *cf*; Figures 5D–5F). As with CPA, 6 mM glucose remained inhibitory for both [Ca^2+^]_i_ spike frequency and the percentage of active α-cells (Figures 6A–6C). In contrast to this, Xestospongin C, an IP_3_R blocker, exerted no inhibitory effect on α-cell [Ca^2+^]_i_ activity or glucagon secretion, when applied at a concentration of 10 μM (Figures S6A–S6D). To directly measure the impact of ryanodine on α-cell ER luminal Ca^2+^ ([Ca^2+^]_ER_) dynamics, Gcg-GCaMP6f mouse islets were transduced with an adenoviral vector containing a fluorescent ER-tagged Ca^2+^ sensor (RCEPIA1er) that reports [Ca^2+^]_ER_.^39^ As shown in Figures 6D and 6H, under control conditions, lowering the extracellular glucose concentration from 6 to 1 mM led to a decline in α-cell [Ca^2+^]_ER_ that was partially reversed by the re-introduction of 6 mM glucose. By comparison, in islets pretreated with ryanodine, α-cell [Ca^2+^]_ER_ release in response to a fall in extracellular glucose concentration was strongly reduced (to ∼20% of the control) (Figures 6E and 6H). Consistent with this Ca^2+^ imaging data, ryanodine inhibited low glucose-stimulated glucagon secretion (Figure 6I). Taken together, these data demonstrate that glucagon secretion during hypoglycaemia requires Ca^2+^ release from the ER via RyRs.

Activation of RyRs, in many cell types, is triggered by opening of Ca_v_ channels and subsequent increase in local [Ca^2+^]_i_, resulting in amplification of the signal by the process of Ca^2+^-induced Ca^2+^ release (CICR).^40^^,^^41^^,^^42^ We tested whether any specific Ca_v_ channels are responsible for CICR in α-cells. In the presence of isradipine, hypoglycaemia-induced [Ca^2+^]_ER_ release was stimulated, albeit the effect was small (Figures 6F and 6H). In contrast, this [Ca^2+^]_ER_ release was abolished by the application of the P/Q-type Ca_v_ channel blocker ω-agatoxin IVA (Figures 6G and 6H). Interestingly, when applied together with ryanodine, ω-agatoxin IVA exerted no additive effect on low glucose-stimulated glucagon secretion (Figure 6I), indicating the P/Q-type Ca_v_ channels may be coupled to CICR in α-cells. Indeed, as shown in Figure 6J, electrical stimulation (a 500-ms depolarization from −70 to 0 mV) of α-cells evoked large Ca^2+^ transients that continued to increase beyond the duration of the depolarizations. This correlated with a slow-component in α-cell exocytosis after the stimuli were withdrawn. Application of ω-agatoxin IVA strongly suppressed depolarization-triggered Ca^2+^ transients (−57% in area under the curve (AUC)) and exocytosis (−65%), with both confined to the duration of the depolarizations (Figures 6K–6M). As such, our data strongly suggest that the opening of P/Q-type Ca_v_ channels triggers CICR in α-cells, providing sufficient Ca^2+^ for efficient stimulation of glucagon secretion.

## Discussion

Glucagon is a potent stimulator of hepatic gluconeogenesis and plays a key role in the maintenance of systemic glucose homeostasis, preventing hypoglycaemia.^43^ Secretion of the hormone is tightly regulated by the blood glucose level, however, exactly how this alters α-cell activity and glucagon secretion remains to be established. In this study, using a combination of electrophysiology, live-cell imaging, and islet hormone secretion, we studied the dynamics of α-cell [Ca^2+^]_i_ and its relationship with glucose-dependent glucagon secretion. Our data reveal that glucose metabolism regulates glucagon secretion via a membrane potential-independent mechanism, exerting its regulatory effect on α-cell [Ca^2+^]_i_ through the control of ER Ca^2+^ release. We propose that P/Q-type Ca_v_ channel-coupled ER Ca^2+^ release, through RyRs, is the key signal that triggers glucagon secretion at low glucose.

α-cells are electrically excitable, active across different concentrations of glucose.^8^ This excitability is enabled by the low conductance of K_ATP_ channels within the cell, even in the presence of low glucose levels, thus the α-cell membrane potential is set at a level that allows opening of voltage-gated ion channels and generation of action potentials.^8^^,^^44^^,^^45^^,^^46^ It has long been documented that α-cell electrical activity is Ca^2+^-dependant, whereas the removal of Na^+^ ^46^ or blockade of Na_v_ channels, with tetrodotoxin,^47^ does not prevent action potential firing (but changes the shape). This pace-making Ca^2+^ conductance was previously proposed to be the low-voltage activated T-type Ca_v_ channel.^8^^,^^45^ Here, our data show that this role, in mouse α-cells, is played by an isradipine-sensitive L-type Ca_v_ channel, similar to what was suggested for human α-cells.^18^ It is reasonable to speculate that this is the Ca_v_1.3 channel (encoded by *Cacna1d*), as it is present in α-cells^17^ and can activate at negative membrane potentials.^48^

Given its striking impact on α-cell electrical activity, it is surprising that isradipine exerts no inhibitory effect on glucagon secretion or α-cell [Ca^2+^]_i_ oscillations at low glucose, an observation consistent with previous studies.^9^^,^^17^^,^^20^^,^^21^^,^^29^^,^^49^ Interestingly, isradipine exerted a stimulatory effect on α-cell [Ca^2+^]_i_ activity when applied at the maximum inhibitory concentration, at both low and high glucose. Although this correlated with a significant increase in glucagon secretion at low glucose, it was not apparent at high glucose, consistent with recent reports demonstrating that α-cell exocytosis is glucose-dependent.^50^^,^^51^ We propose that any potential isradipine-dependent inhibition of glucagon secretion may be masked by a reduction in intra-islet somatostatin paracrine signaling. This is supported by the data that isradipine significantly inhibited somatostatin secretion at low glucose (Figure S2A) and had no additive effect on α-cell Ca^2+^ activity when applied in the presence of CYN (a blocker of SSTR in α-cells) (Figure S2F). Furthermore, like SSTR blockers, isradipine induced an increase in cytosolic cAMP (Figure S3C). This may contribute to the elevated glucagon secretion (Figure 1D by 1) potentiating ER Ca^2+^ mobilization (Figures 6F and 6H), in a manner similar to that described in δ-cells^42^ and 2) enhancing α-cell exocytosis.^49^

We observed that isradipine strongly inhibits α-cell electrical activity, without affecting glucagon secretion, and this suggests that glucagon release can be independent of α-cell electrical activity (Figures 1C and 1D). This may explain the observation that isradipine reduced the dependence of glucagon secretion on P/Q-type Ca_v_ channels, the opening of which requires action potential firing^10^ (Figure 1D). Importantly, this also suggests the presence of an intracellular source that provides the Ca^2+^ required for exocytosis/glucagon secretion. Our pharmacological analyses identified that this role is played by the ER (Figure 5). The ER is an important organelle that bears multiple vital biological functions, including the maintenance of intracellular Ca^2+^ homeostasis (reviewed in Daverkausen-Fischer and Pröls^52^). It is the major intracellular Ca^2+^ store and can be leaky.^53^ It also can function as a high-affinity Ca^2+^ sink that rapidly sequesters cytosolic Ca^2+^. In β cells, the ER has been shown to be regulated by glucose or intracellular ATP.^38^ Metabolic sensing by the ER is also likely to operate in α-cells and may account for the glucose-dependent suppression of α-cell [Ca^2+^]_i_ oscillations and glucagon secretion in the presence of isradipine (i.e., in the absence of electrical activity). Therefore, although similar to isradipine in blocking α-cell electrical activity, diazoxide’s strong repolarizing effect can reduce Na^+^/K^+^ pump activity, leading to an increase in the intracellular ATP concentration, via a “sparing effect” (while isradipine does not) (Figures 3F–3M). We reason that this may account for its ability to block low-glucose-stimulated glucagon secretion and α-cell [Ca^2+^]_i_ activity (Figures 3A–3D).

At low glucose, reduced cytosolic ATP restricts Ca^2+^ loading into the ER (due to the reduced action of the ATP-consuming SERCA Ca^2+^-ATPase) and sustained release of ER Ca^2+^ was observed (Figure 6). This correlates with the isradipine-resistant spontaneous α-cell [Ca^2+^]_i_ oscillations (Figure 2) and could be induced by intracellular application of low ATP (Figure 4). As the rate of glucagon secretion was not reduced when membrane electrical activity was absent, Ca^2+^ mobilized from the ER is evidently sufficient to trigger α-cell exocytosis and glucagon secretion. By comparison, electrical activity-dependent Ca^2+^ influx alone was insufficient to evoke glucagon release, although it too is controlled by glucose metabolism (Figures 5B, 5C, 6B, and 6C). Blockade of electrical activity-mediated Ca^2+^-influx, with isradipine or agatoxin, exerted little additional impact on glucagon secretion when applied in the presence of CPA or ryanodine (Figures 5H, 5I, 6H, and 6I). Our pharmacological analyses show that RyRs, the ER-bound Ca^2+^-release channels previously reported to contribute to adrenaline-stimulated glucagon secretion,^49^ are the channels responsible for this Ca^2+^ signal (Figures 4 and 6). While the activation of RyRs normally requires transmembrane Ca^2+^ influx, initiated during the firing of action potentials, our data show ER Ca^2+^ release is sustained in low glucose when α-cell electrical activity was abolished (Figure 6). This is consistent with the ryanodine-sensitive Ca^2+^ mobilization observed in the presence of low ATP (Figure 4). Exactly how low glucose metabolism activates RyRs remains to be established. It is possible that the elevation of α-cell intracellular cAMP at low glucose^32^ may induce spontaneous Ca^2+^ release via RyRs, a mechanism described in pancreatic δ-cells.^42^ Interestingly, when α-cell electrical activity was intact, release of ER Ca^2+^ via activation of RyRs was dependent on the opening of P/Q-type Ca_v_ channels (Figures 6G, 6H, and 6J–M). Together these may explain the observations that low glucose enhances depolarization-triggered exocytosis in α-cells.^50^^,^^51^

In conclusion, based on the data presented in this study, we propose a new model of glucose regulated glucagon secretion (illustrated in the graphical abstract). At low extracellular glucose concentrations, a drop in intracellular ATP leads to partial opening of K_ATP_-channels on the membrane, enabling generation of large overshooting action potentials. These action potentials can open P/Q-type Ca_v_ channels that, in turn, activate RyRs on the ER to trigger CICR, stimulating glucagon secretion. At high extracellular glucose concentrations, the impact of elevated intracellular ATP is 2-fold: (1) it closes the K_ATP_ channels, depolarizing the α-cell membrane, reducing the action potential amplitude to a level that cannot open the P/Q-type Ca^2+^ channels; and (2) it enhances Ca^2+^ sequestration into the ER, and in combination with lower RyR activity, α-cell [Ca^2+^]_i_ activity and glucagon secretion is reduced. As such, in addition to its role in α-cell electrical activity,^11^ the ER provides “double security” by controlling α-cell [Ca^2+^]_i_: at low glucose, it provides sufficient Ca^2+^for α-cell exocytosis, meeting the demands for high-volume secretion to restore euglycaemia; at high glucose, it sequesters excess Ca^2+^ that might lead to unwanted glucagon secretion. Consequently, secretion of these important hyperglycaemic hormones remains tightly controlled for optimal systemic glycemic regulation.

### Limitations of the study

Glucagon secretion is subject to both intrinsic and paracrine regulation.^54^ The current study focused on the intrinsic aspect of α-cell physiology without detailing the impact of paracrine factors on ER-dependent glucagon secretion. The effects of ER modulators were mainly observed in the presence of low glucose (1 mM; Figures 5H, 6H, and 6I), a condition that has been reported to be associated with minimal activity in neighboring β and δ-cells.^54^ Therefore, it is unlikely that effects observed were due to changes in intra-islet paracrine signaling, but rather reflect the direct impacts of these modulators on α-cells. The high glucose in this study refers 6 mM glucose, which is the maximum inhibitory concentration for glucagon secretion^54^^,^^55^ and is consistent with the experimental conditions used in our previous studies.^10^^,^^33^^,^^56^ We therefore did not test the effect of glucose >6 mM and cannot rule out that higher glucose concentrations may have additional effects on α-cell physiology (e.g., activation of β cells may alter the paracrine control of glucagon secretion^57^).

Another limitation of this study is that all experiments were performed on isolated mouse islets and may not directly translate to human islets or *in vivo*. First, in the body, α-cell ER Ca^2+^ release can also be stimulated by increased adrenergic signaling in the circulation,^49^ occurring normally during hypoglycaemia.^58^ As such, the combined effects of low glucose and adrenaline can produce a robust glucagon response, facilitating rapid recovery from hypoglycaemia. Second, mouse and human pancreatic islets share many similarities^59^ but α-cell ion channel composition does differ.^18^ However, the function of the L-type Ca_v_ channel is conserved in human α-cells and is involved in the generation of action potentials.^18^ We note that isradipine exerts a stronger effect on human than on mouse α-cells, but it does not abolish either Ca^2+^ activity or low-glucose-stimulated glucagon secretion.^9^^,^^18^ Therefore, it is highly likely an intracellular Ca^2+^ source, as described here in mouse α-cells, may also be involved in glucagon secretion from human α-cells.

For parallel recordings of two fluorescent signals (e.g., Perceval and Calbryte-630), a widefield microscope (AxioZoom) was used in this study. This can result in background noise from out-of-focus signals when intact islets were used. However, the adenovirally delivered probes typically express only on the periphery of the islets and the islets are normally slightly flattened against the coverslip in the recording chamber. These experimental conditions, together with limiting the size of regions of interest during analysis, were effective in minimizing noise. Furthermore, recordings from intact islets and dispersed monolayer cells showed identical effects.

## STAR★Methods

### Key resources table

**Table:** 

REAGENT or RESOURCE	SOURCE	IDENTIFIER
**Antibodies**		

rabbit FITC 495-conjugated anti-GFP	InsightBio	Cat# DS-PB-00926; RRID:AB_854001
mouse anti-glucagon	Sigma-Aldrich	Cat# G2654; RRID:AB_259852
guinea-pig anti-insulin	Dako	Cat# A0564; RRID:AB_10013624
goat anti-somatostatin	Santa Cruz Biotechnology	Cat# sc-7819; RRID:AB_2302603
Alexa Fluor 633 goat anti-mouse IgG	ThermoFisher	Cat# A-21052; RRID:AB_2535719
Alexa Fluor 594 goat anti-guinea pig IgG	ThermoFisher	Cat# A-11076; RRID:AB_2534120
Alexa Fluor 546 donkey anti-goat IgG	ThermoFisher	Cat# A-11056; RRID:AB_2534103

**Chemicals, peptides, and recombinant proteins**		

tamoxifen	Sigma-Aldrich	Cat# T5648
adrenaline	Sigma-Aldrich	Cat# E4375
Liberase T-Flex	Roche	Cat# 9001-12-1 and 9073-78-3
Fluo-4 pentapotassium	ThermoFisher	Cat# F14200
cyclopiazonic acid	Sigma-Aldrich	Cat# C1530
Calbryte 520-AM	AAT Bioquest	Cat# 20651
Calbryte 630-AM	AAT Bioquest	Cat# 20721
Isradipine	Alamone	Cat# I-100
Ryanodine	Bio-techne	Cat# 1329/1
ω-agatoxin IVA	Tocris	Cat# 2799
ω-conotoxin GIVA	Alomone	Cat# C-300
Tetrodotoxin	Alomone	Cat# T-550
BayK8644	Tocris	Cat# 1544
CYN 154806	Bio-Techne	Cat# 1843/1
Xestospongin C	Abcam Plc	Cat# ab120914
Diazoxide	Merck	Cat# D9035

**Critical commercial assays**		

Glucagon ELISA	Mercodia AB	10-1271-01

**Experimental models: Organisms/strains**		

Mouse: C57BL/6J	Jackson Laboratory	RRID:MGI:3028467
Mouse: Glu^Cre/ERT2^	Jackson Laboratory	RRID:MGI:J:236327
Mouse: Rosa26^GCaMP6f^	Jackson Laboratory	RRID:MGI:028865

**Recombinant DNA**		

R-CEPIA1er plasmid	Suzuki et al.^39^	AddGene: #58216
GW1CMV-Perceval plasmid	Berg et al.^34^	AddGene: #21737

**Software and algorithms**		

ZEN Black – Version 6.0.0.303	Zeiss	RRID:SCR_018163
μManager – Version 2.0.0	Ron Vale laboratory	RRID:SCR_000415
FIJI (ImageJ) – Version 1.53c	National Institute of Health	RRID:SCR_002285; https://imagej.net/software/fiji/
Clampfit – Version 10.7.0.3	Molecular Devices	RRID:SCR_011323
Prism – Version 9.5.1	GraphPad	RRID:SCR_002798
Pulse – Version 8.80	HEKA Electronics	RRID:SCR_025158

### Resource availability

#### Lead contact

Further information and reasonable requests for resources and reagents should be directed to and will be fulfilled by the lead contact, Quan Zhang (quan.zhang@ocdem.ox.ac.uk).

#### Materials availability

This study did not generate new unique reagents.

#### Data and code availability

• All data reported in this paper will be shared by the lead contact upon reasonable request.
• This paper does not report original code
• Any additional information required to reanalyze the data reported in this paper is available from the lead contact upon request.

### Experimental model and study participant details

#### Animals and islet isolation

All animal experiments were conducted in accordance with the UK Animals Scientific Procedures Act (1986) and the University of Oxford ethical guidelines. Mice were kept on a 12-h light-dark cycle and allowed free access to chow diet and water. For hormone secretion and electrophysiological studies, female C57B/6J (RRID:MGI:3028467; Envigo) mice >15 weeks old were used. Most Ca^2+^ imaging experiments used transgenic reporter mouse model expressing a genetically encoded high affinity (Kd = 600nM) calcium indicator GCaMP6-fast variant (GCaMP6f)^60^ specifically in α-cells (Gcg-GCaMP6f). The mouse model was generated using a Cre-LoxP approach by crossing a mouse line that expresses a tamoxifen-inducible Cre recombinase in glucagon-expressing cells (Glu^Cre/ERT2^ mouse line^61^; RRID:MGI:J:236327, Jackson Laboratory, Bar Harbor, Maine, USA), with a floxed GCaMP6f mouse line (Rosa26^GCaMP6f^ mouse line^62^; Ai95D, RRID:MGI:028865, Jackson Laboratory). The offspring, double transgenic animals (Glu^Cre/ERT2^;GCaMP6f, or Gcg-GCaMP6f), demonstrated a high recombination rate and α-cell specific expression of GCaMP6f in islets (95 ± 3% of glucagon positive cells; none in insulin positive cells; *n* = 5 islets; Figure S1A). α-cell Cre-dependant recombination was induced by a regimen of oral gavage with tamoxifen (20 mg/mL in corn oil; T5648; Sigma-Aldrich, St. Louis, Missouri, USA) for 5 consecutive days. The function of GCaMP6f, as a reporter of α-cell intracellular Ca^2+^, was confirmed by a rapid increase in the reporter fluorescence in response to adrenaline (5 μM)^49^(Figure S1B).

Mice were sacrificed by cervical dislocation, with pancreas extraction following intra-ductal injection with 150 μg/mL liberase and 7.5 μg/mL thermolysin mix (Liberase Flex, Roche, Basel, Switzerland) in Hank’s Balanced Salt Solution (Sigma). Islets were hand-picked following enzymatic digestion and cultured in RPMI 1640 medium (Life Technologies, Carlsbad, California, United States) supplemented with 1% penicillin/streptomycin (Gibco), 5 mM glucose, and 10% fetal bovine serum (Sigma-Aldrich), at 37°C and 5% CO_2_, before experimentation.

Both sexes of the animals were used in this study. No sex-dependent differences were observed.

### Method details

#### Chemical compounds studied in this article

Isradipine (PubChem CID: 3784); Cyclopiazonic acid (PubChem CID: 135494311), Diazoxide (PubChem CID: 3019); Ryanodine (PubChem CID: 11317883); Xestospongin C (PubChem CID: 5311502); ω-agatoxin IVA (PubChem CID: 56841669); ω-conotoxin GVIA (PubChem CID: 16132374); Tetrodotoxin (PubChem CID: 11174599); CYN154806 (PubChem CID: 16133842).

#### Imaging of cytoplasmic Ca^2+^

Islets from male and female Gcg-GCaMP6f mice (>15 weeks) were used for most live-cell Ca^2+^ imaging experiments. Islets were immobilised on an 18 mm poly-L-Lysine coated coverslip fixed in a custom-built imaging chamber filled with Krebs-Ring buffer (KRB) consisting of 140 mM NaCl, 3.6 mM KCl, 0.5 mM MgSO_4_, 2.6 mM CaCl_2_, 0.5 mM NaH_2_PO_4_, 2 mM NaHCO_3_, and 5 mM HEPES (pH 7.4, adjusted using NaOH), at 6 mM glucose, for 10 min prior to experiments. These were performed using an inverted LSM 510 confocal microscope (Zeiss) controlled with ZEN Black (Zeiss), using a 40×/1.3 Oil immersion objective. Time-lapse images were collected every 0.98211 s with a frame size of 256 × 256 pixels and the bath solution was heated at 37°C, perfused at 400 μL/min. GCaMP6f was excited by an argon laser (488 nm) and emission were collected at 510 nm.

Data in Figures 4 and 6J–6M were generated by combination of electrophysiology and [Ca^2+^]_i_ imaging. In this series of experiments, [Ca^2+^]_i_ was monitored using Fluo-4 pentapotassium (ThermoFisher) injected into the cell through a patch pipette. Patch pipettes were filled with intracellular solution consisting of: 125 mM Cs-glutamate, 10 mM CsCl, 10 mM NaCl, 1 mM MgCl_2_, 0.05 mM EGTA, 3 mM Mg-ATP, 0.1 mM cAMP, 5 mM HEPES and 25 μM Fluo-4 pentapotassium (pH 7.1 using CsOH). Fluo-4 was excited using an LED light source (wLS LED Illumination Unit, QImaging) and emitted light passed through a FITC filter set (Nikon) before being detected by a sCMOS camera (OptiMOS, QImaging) controlled by μManager (Ron Vale laboratory, UCSF).

#### Imaging of endoplasmic reticulum Ca^2+^

For simultaneous monitoring of intracellular and intraluminal ER Ca^2+^, tamoxifen-induced islets from male and female Gcg-GCaMP6f mice (>15 weeks) were infected with an adenovirus that carries the construct encoding the double inverted orientation (DIO) ER-tagged low affinity probe (K_d_ = 370 μM) RCEPIA1er^39^ in 50 μL droplets of complete RPMI medium and incubated at 37°C and 5% CO_2_ for 36 h. Viral transduction was halted by the transfer of islets into virus-free RPMI culture medium. The ability of RCEPIA1er to report ER intraluminal free Ca^2+^ is validated by cyclopiazonic acid (CPA), which induced a rapid decline of RCEPIA1er fluorescent intensity, reflecting depleting of the ER The ability of RCEPIA1er to report ER intraluminal free Ca^2+^ is validated by cyclopiazonic acid (CPA), which induced a rapid decline of RCEPIA1er fluorescent intensity, reflecting depleting of the ER^63^ (Figure S3). In some experiments, an organic Ca^2+^ indicator was used together with RCEPIA1er. Islets were loaded with 1 μM of the Ca^2+^-indicator Calbryte 520-AM (AAT Bioquest, Pleasanton, California, USA) for 1 h before imaging.

Islets were first stabilised with KRB supplemented with 6 mM glucose in a microperifusion imaging chamber^64^ for 10 min prior to experiments. Ion channel modulators and glucose were applied as indicated. Signals were imaged using a Zeiss AxioZoom.v16 system equipped with a 2.3×/0.56 objective (Zeiss). The fluorescent reporters were co-imaged from the same cell in a single-wavelength mode, the excitation/emission wavelength being (nm): 490/535 (GCaMP6f or Calbryte 520), 532/588 (RCEPIA1er). Cell images were acquired with a large-matrix (3216 × 2208) CCD AxioCam 807 with a pixel size of 4.5 × 4.5 μm^2^, peak QE of 78% and full well capacity of 25,000 e, every 10 s at 37°C, perfused at 58 μL/min. No effects of phototoxicity were recorded during the experiments.

#### Imaging of intracellular ATP/ADP

In order to measure the ATP/ADP of α-cells, islets were isolated from female C57/B6J mice (>15 weeks) and infected with an adenovirus carrying the ATP/ADP probe, Perceval,^34^ in a 50 μL virus-containing droplet and incubated at 37°C, 5% CO_2_ for 24 h. Subsequently, islets were loaded with 1 μM of the Ca^2+^-indicator Calbryte 630-AM (AAT Bioquest, Pleasanton, California, USA) for 1 h before being loaded into a custom-made imaging chamber.^64^ Islets were perfused with KRB containing 1 mM glucose, for 10 min prior to experiments. Signals were imaged using a Zeiss AxioZoom.v16 system equipped with a 2.3×/0.56 objective. The fluorescent reporters were co-imaged from the same cell in a single-wavelength mode, the excitation/emission wavelength being (nm): 490/535 (Perceval), 532/588 (Calbryte 630). Cell images were acquired every 10 s at 37°C, perfused at 132 μL/min α-cells were identified by positive Ca^2+^ response to 10 μM adrenaline. No effects of phototoxicity were recorded during the experiments and cells responded to FCCP (by showing a rapid drop in Perceval signal) at the end of the recordings.

#### Imaging of intracellular cAMP

To measure [cAMP]_i_ in α-cells, isolated islets were transfected with a genetically encoded green fluorescent Upward cAMP sensor, baculovirus-carrying cADDis (Montana Molecular, Bozeman, Montana, United States), in a 50 μL droplet at 37°C and 5% CO_2_ for 36 h before imaging. Islets were subsequently loaded into a microperfusion imaging chamber^64^ and stabilised with KRB containing with 1 mM glucose for 10 min prior to experiments. Signals were imaged using a Zeiss AxioZoom.v16 system equipped with a 2.3×/0.56 objective. The fluorescent reporters were imaged in single-wavelength mode, the excitation/emission wavelength being (nm): 490/535. α-cells were functionally identified by their responses to adrenaline (increases). Cell images were acquired every 10 s at 34°C, perfused at 134 μL/min.

#### Measurements of glucagon secretion

Glucagon secretion was performed by static incubation as previously described.^65^ 15–20 size-matched isolated islets were placed into microfuge tubes and pre-incubated for 60 min at 37°C in 150 μL KRB supplemented with 3 mM glucose; pharmacological agents were added as indicated. Media was subsequently replaced with testing KRB buffer supplemented with glucose and/or pharmacological agents, as indicated. After a 60 min incubation at 37°C, the supernatant was removed and stored at −80°C before being assayed using a glucagon enzyme-linked immunosorbent assay (glucagon ELISA, 10-1271-01, Mercodia AB, Uppsala, Sweden). For content measurements, acidic ethanol was added to islets before sonication for content extraction, and storage at −20°C prior to measurement by glucagon ELISA (Mercodia).

#### Electrophysiology

All electrophysiological measurements were performed using an EPC-10 patch clamp amplifier and Pulse software (version 8.80, HEKA Electronics). Electrical activity, membrane currents and changes in cell capacitance (reflecting exocytosis) were recorded from superficial α-cells in intact, freshly isolated mouse pancreatic islets^20^ using the perforated patch or standard whole-cell techniques as indicated. Patch pipettes were pulled from borosilicate glass with resistances of ∼5 MΩ when filled with the pipette solutions. Whole cell access was achieved either by rupturing the cell membrane within the patching electrode (for standard whole cell; with series resistance, Rs, of <30 MΩ) or by amphotericin B (perforated technique). All electrophysiological measurements were carried out at 32°C–34°C. The α-cells were identified by electrophysiological fingerprinting.^26^ Exocytosis was detected as changes in cell capacitance, estimated by the Lindau-Neher technique implementing the ‘Sine+DC’ feature of the lock-in module of the Pulse software. The sine wave amplitude was 20 mV and the frequency was 1250 Hz.^66^

For membrane potential recordings, the perforated patch technique was used on C57/B6J. The pipette solution contained 76 mM K_2_SO_4_, 10 mM NaCl, 10 mM KCl, 1 mM MgCl_2_ and 5 mM HEPES (pH 7.35 with KOH). Membrane perforation was achieved by inclusion of amphotericin B (0.24 mg/mL) in the pipette-filling solution. For standard whole cell experiments, the intracellular solution contained: 125 mM Cs-glutamate, 10 mM CsCl, 10 mM NaCl, 1 mM MgCl_2_, 0.05 mM EGTA, 3 mM Mg-ATP, 0.1 mM cAMP and 5 mM HEPES (pH 7.1 using CsOH). For ATP infusion experiments, cAMP was excluded and intracellular ATP concentrations are as indicated. KRB was used as the extracellular solution with glucose concentrations as indicated in the figures.

#### Immunofluorescence staining

Immunostaining was conducted on isolated islets, fixed in 4% paraformaldehyde before being permeabilised using 0.1% Triton X-100 (Sigma). After blocking with 5% goat serum, islets were incubated overnight (4°C) with primary antibodies before incubation with secondary antibodies. Fluorescent staining was visualised using a laser-scanning confocal microscopy (BioRad) controlled by LaserSharp2000 (BioRad).

Antibodies used in this study were: rabbit FITC 495-conjugated anti-GFP (1:250; DS-PB-00926; InsightBio, Wembley, United Kingdom), mouse anti-glucagon (1:1000; G2654; Sigma), guinea-pig anti-insulin (1:400; A0564; Dako, Santa Clara, California, United States), goat anti-somatostatin (1:100; sc-7819; Santa Cruz Biotechnology, Dallas, Texas, United States); Alexa Fluor 633 goat anti-mouse IgG (1:500; A-21052; ThermoFisher); Alexa Fluor 594 goat anti-guinea pig IgG (1:500; A-11076; ThermoFisher); and Alexa Fluor 546 donkey anti-goat IgG (1:100; A-11056; ThermoFisher).

### Quantification and statistical analysis

Imaging videos were analyzed using Fiji imaging processing package (1.50days, National Institute of Health). Region of interest (ROI) size was minimised to reduce the influence of side and out of focus fluorescence. The mean fluorescence (F) of each ROI was normalised to baseline signal (F_0_) and expressed as F/F_0_ before exporting into ClampFit (9.2.0.11, Molecular Devices), where area under the curve (AUC), peak signal amplitude, and spike frequency were calculated. For RCPEIA1er, AUC was calculated during the first 3.5 min from the initial deflection from the baseline level at 6 mM glucose.

For membrane potential recordings, data were exported from the Pulse and converted into axon binary files (ABF) using ABF File Utility (v2.1.57, Synaptosoft Inc., Fort Lee, NJ) before being analyzed in ClampFit (9.2.0.11, Molecular Devices, USA). Cell exocytosis (detected using capacitance measurements) and transmembrane currents were measured manually using Pulse.

All data are reported as mean values ±standard error of the mean (SEM), unless otherwise stated. For two groupings, a Student’s t test was conducted. For secretion experiments, Fisher’s least significant difference test was utilised. Outliers were identified by ROUT (Q = 1%). ‘n’ is defined as the number of technical replicates, unless otherwise specified. All statistical analyses were performed using GraphPad Prism (version 9.5.1) and statistical significance was defined as *p* < 0.05.

## Supplemental information

Document S1. Figures S1‒S6
