# Supplementary material for: The endoplasmic reticulum plays a key role in α-cell intracellular Ca2+ dynamics and glucose-regulated glucagon secretion in mouse islets
Source: iScience. 2024 Apr 5;27(5):109665. doi: 10.1016/j.isci.2024.109665 (PMC11033163; doi:10.1016/j.isci.2024.109665)
Supplement: Document S1. Figures S1‒S6 [file mmc1.pdf]

iScience, Volume *27*

Supplemental information

The endoplasmic reticulum plays a key role in α-cell intracellular Ca^{2+} dynamics and glucose-regulated glucagon secretion in mouse islets

Samuel Acreman, Jinfang Ma, Geoffrey Denwood, Rui Gao, Andrei Tarasov, Patrik Rorsman, and Quan Zhang

Supplementary Information

Supplementary Figures and Legends

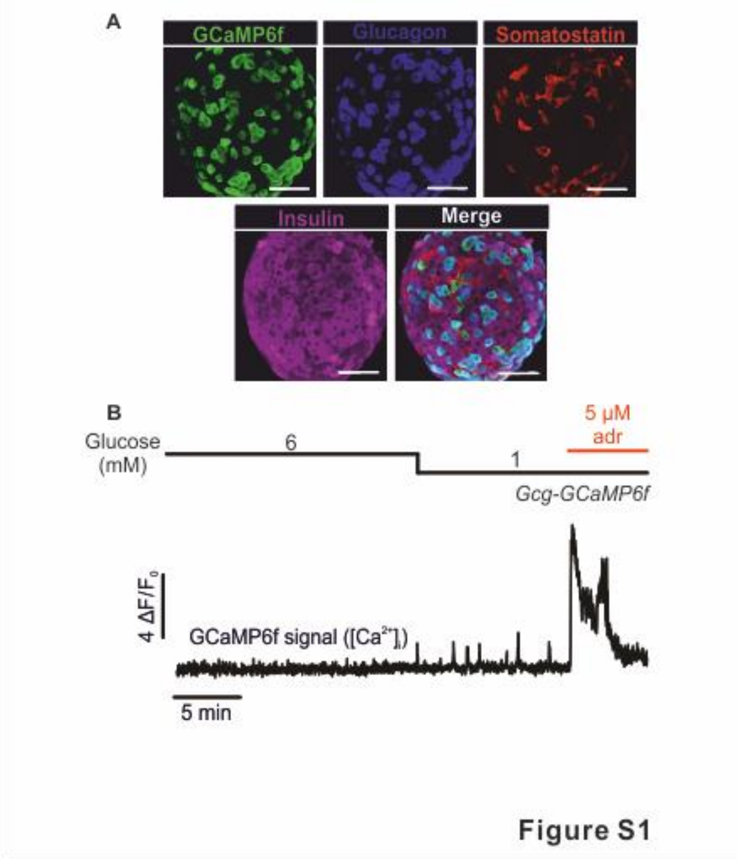

Figure S1. The expression of GCaMP6f and functional $[Ca^{2+}]_i$ response in islets from the Gcg-GCaMP6f mouse model. Related to Figure 2.

(A) Immunofluorescence staining of a Gcg-GCaMP6f islet with GFP (GCaMP6f, green), glucagon (blue), somatostatin (red) and insulin (purple), as well as a merged slide to show co-localisation. **(B)** Representative trace showing functional response of GCaMP6f to a reduction of glucose from 6 to 1 mM and the addition of 5µM adrenaline (Adr) in Gcg-GCaMP6f islets.

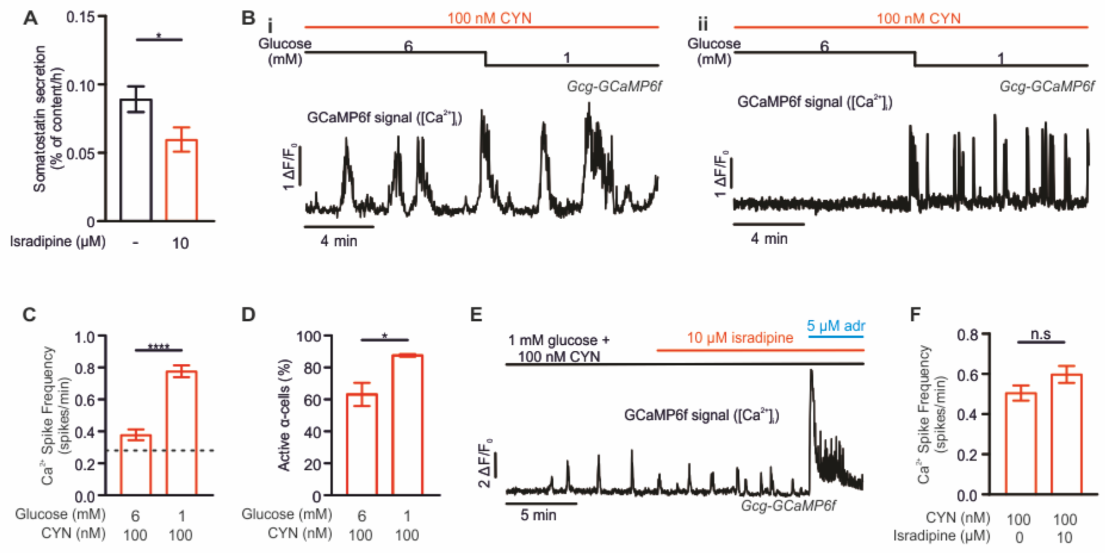

Figure S2

Figure S2. Comparable effects of isradipine and CYN on α-cells. Related to Figure 2.

(A) Somatostatin secretion secretory response from intact islets at 1 mM glucose in the absence (black bar; n=4) or presence (red bar; n=4) of 10 µM isradipine. **(B)** Representative traces showing the α-cell $[Ca^{2+}]_i$ response, as measured by Gcg-GCaMP6f fluorescence, to changes in extracellular glucose from 6 to 1 mM in the presence of 100 nM CYN. Both fast (i) and slow (ii) oscillations were observed. **(C)** Bar graph summarising the frequency of spontaneous α-cell $[Ca^{2+}]_i$ spikes to changes in extracellular glucose from 6 to 1 mM in the presence of 100 nM CYN, as in B (dashed line represents the frequency value of α-cells at 1 mM glucose alone, as shown in Fig. 2C; n=250 cells from 5 islets). **(D)** As in C but shows the average fraction of active α-cells under the indicated conditions (n=5 islets). **(E)** Representative trace showing the α-cell $[Ca^{2+}]_i$ response, as measured by GCaMP6f fluorescence, to the addition of 10 µM isradipine in the presence of 100 nM CYN at 1 mM glucose. **(F)** Bar graph summarising the frequency of α-cell $[Ca^{2+}]_i$ spike cells under the indicated conditions, as in E (n=132 cells from 4 islets). Data presented as mean ± SEM. *p<0.05, ****p<0.0001 between indicated groups.

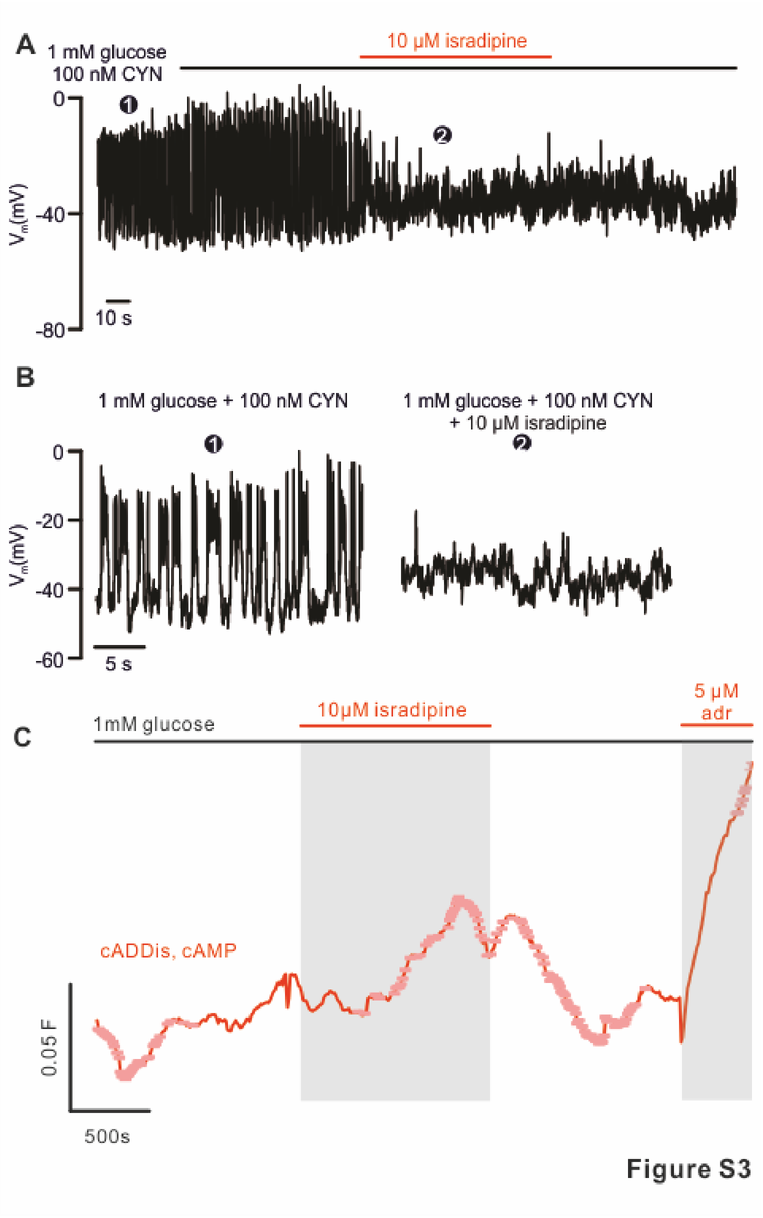

Figure S3. Isradipine effect on α-cell membrane potential in the presence of CYN and on cytosolic cAMP. Related to Figure 2.

(A) Membrane potential recording of an α-cell in the continuous presence of 1 mM glucose and 100 nM CYN. Application of 10 µM isradipine is marked by the horizontal bar over the trace. Data represents 3 recordings. **(B)** Membrane potential recordings, as in A, under (❶) 1 mM glucose and 100 nM CYN and (❷) 1 mM glucose, 100 nM CYN and 10 µM isradipine on expanded time scale. **(C)** Average trace showing the α-cell cAMP response, as measured by cADDis fluorescence, to the addition of 10 µM isradipine and adrenaline (5 µM)at 1 mM glucose.

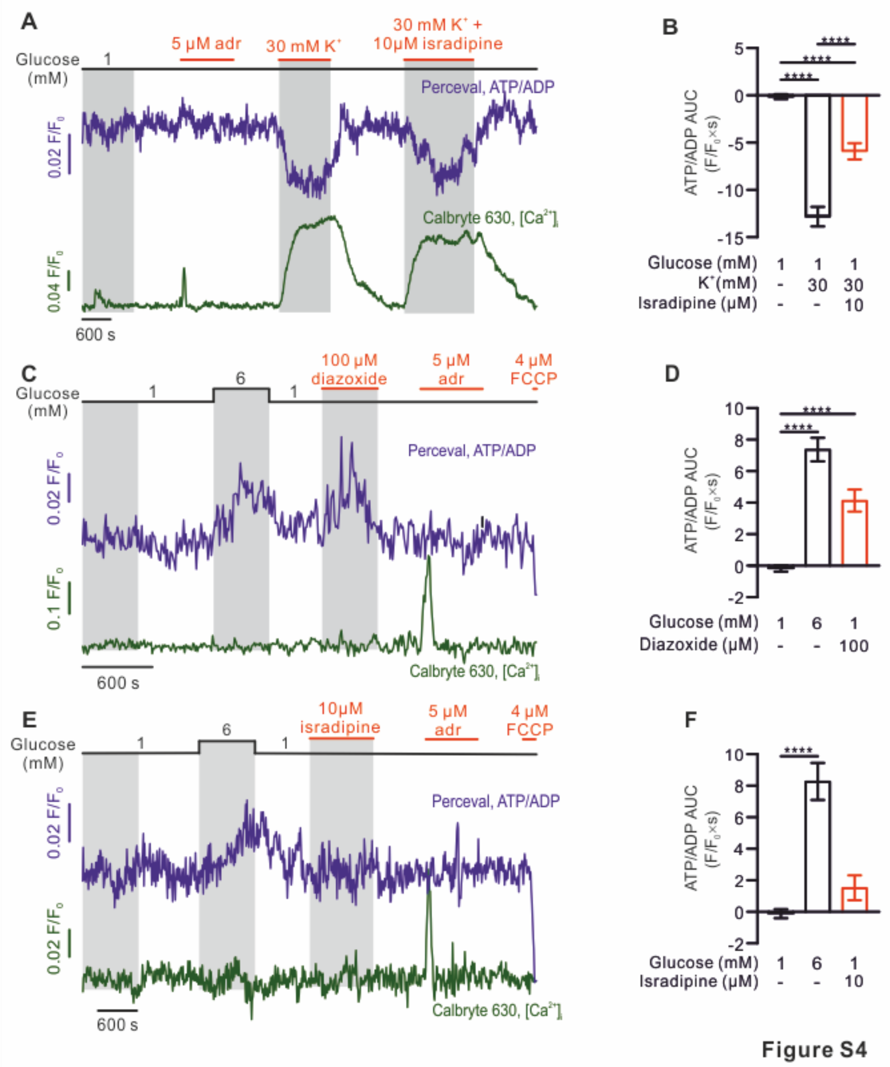

Figure S4. α-cell intracellular ATP/ADP response to high K$^+$-triggered Ca^{2+} influx and in dissociated cell monolayer. Related to Figure 3.

(A) Representative traces showing α-cell intracellular ATP/ADP, as measured by Perceval fluorescence (blue line), and Ca^{2+} (green line), as measured by Calbryte-630 fluorescence, in response to 30 mM K$^+$ with and without the addition of isradipine (10 μM). **(B)** The average AUC of ATP/ADP measured in α-cells under indicated conditions, as in A (n=14). **(C)** As A but shows the response to increasing glucose from 1 to 6 mM and the addition of 100 μM diazoxide in dispersed single α-cells. **(D)** The average AUC of ATP/ADP measured in α-cells under indicated conditions, as in C (n=16). **(E)** As C but shows the response to 10 μM isradipine in dispersed single α-cells. **(F)** As in D but summarises the α-cell ATP/ADP response to isradipine (n= 11), as in C. In all experiments, Adrenaline (adr) was used for functional identification of α-cells and FCCP for evaluating the efficiency of Perceval and cell viability. Cell failed to respond to FCCP were not used for analysis. Condition durations were consistent, as indicated in the grey shaded areas in example traces (A, C and E).

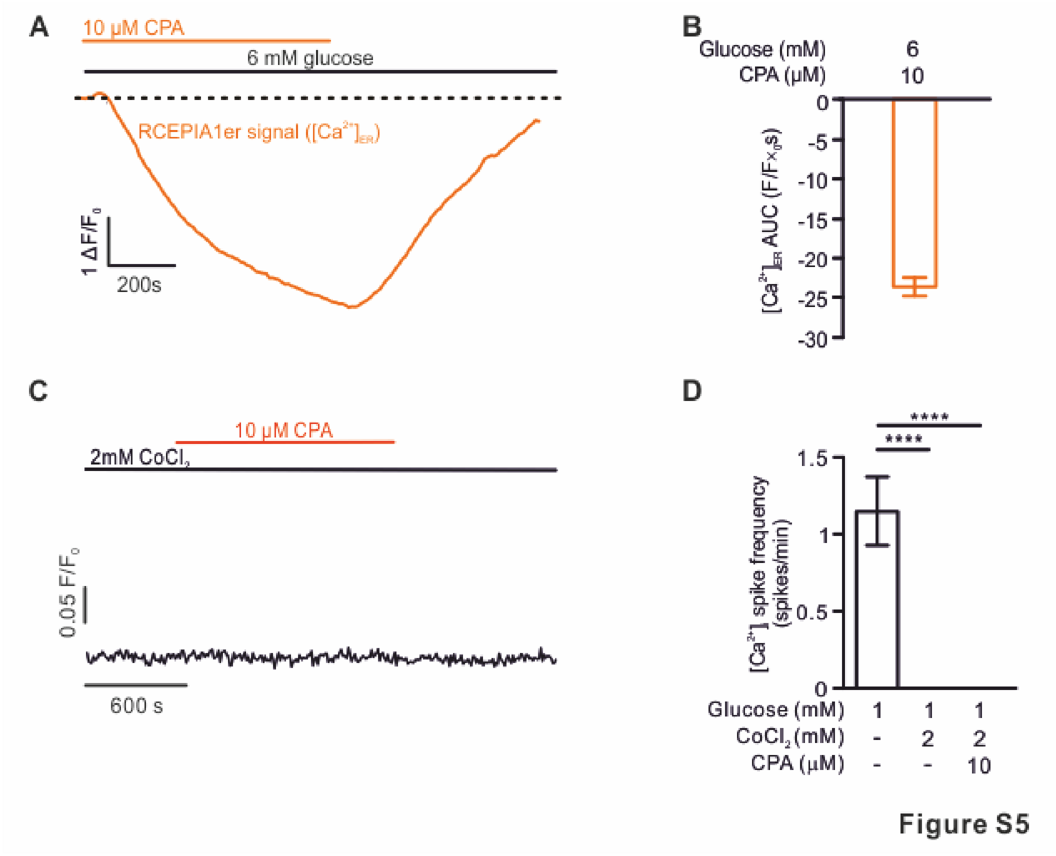

Figure S5. The effect of CPA on α-cell $[Ca^{2+}]_{ER}$ during euglycaemia and on cytosolic Ca^{2+} in the absence of extracellular Ca^{2+}. Related to Figure 5.

(A) Representative trace demonstrating α-cell $[Ca^{2+}]_{ER}$ response, as measured by RCEPIA1er fluorescence (orange) to addition of 10 μM CPA to islets at 6 mM glucose. **(B)** Bar graph summarising α-cell $[Ca^{2+}]_{ER}$ response to 10 μM CPA at 6 mM glucose (orange bar; n=50 cells from 13 islets), as represented in A, measured by AUC of RCEPIA1er fluorescence, for initial 210 seconds of treatment. **(C)** Representative trace showing the α-cell $[Ca^{2+}]_i$ response, as measured by GCaMP6f fluorescence, to the addition of 10 μM CPA in the absence of extracellular Ca^{2+} (when replaced with Co^{2+}, indicated by yellow bar) at 1 mM glucose. Experiments were conducted using single dispersed α-cells. **(D)** Bar graph summarising the frequency of spontaneous α-cell $[Ca^{2+}]_i$ spikes under the indicated conditions, as in C (n=32). Data presented as mean ± SEM. ****p<0.0001 vs control.

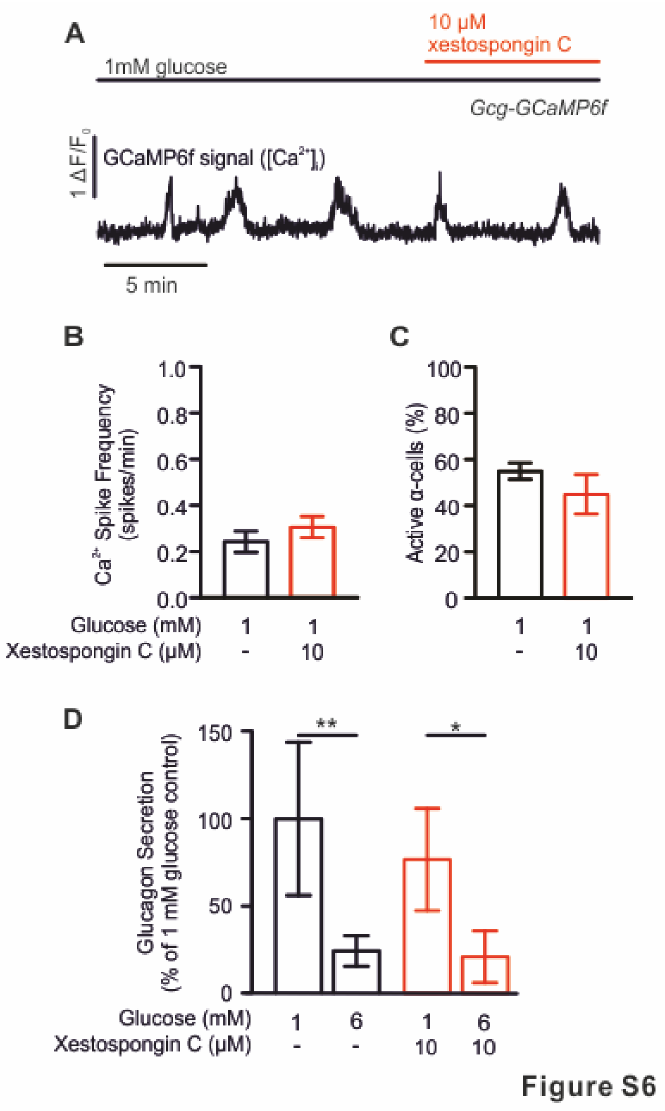

Figure S6. Effect of inhibition of IP_3 receptors on $[Ca^{2+}]_i$ and glucagon secretion. Related to Figure 6.

(A) Gcg-GCaMP6f α-cell $[Ca^{2+}]_i$ response to 10 μM Xestospongin C in the continuous presence 1 mM glucose. The duration of Xestospongin C application is marked by the red horizontal bar. **(B)** Bar graph summarising the frequency of spontaneous α-cell $[Ca^{2+}]_i$ spikes under the indicated conditions (n=134 cells from 4 islets). **(C)** As in B but shows the average fraction of active α-cells following the indicated pretreatment condition (n=4 islets). **(D)** Islet glucagon secretory response to 1 and 6 mM glucose in the absence (black bars; n=4) or presence of 10 μM Xestospongin C (red bars; n=4). Data presented as mean ± SEM. *p<0.05, **p<0.01 between indicated groups.
